# Quick Method of Making Dies

**Published:** 1871-05

**Authors:** J. A. Troutman

**Affiliations:** Toronto.


### ARTICLE VIII.

### *Quick Method of Making Dies.*

By J. A. Troutman, Toronto.

I first take an impression of the mouth and make a model the same as for vulcanite, then take a flask—the same as is used for vulcanite—and drill three or four holes through the bottom; fill the lower part of the flask partially with sand, a little damp; put some sand on the inside of the model, and press or drive the model firmly in the flask, then press the sand around the outside of the model, not allowing it to come up any further than the impression did; brush around the model, and then insert a sharp instrument through the holes in the bottom of the flask to allow the steam to pass out, then lift out the model and replace any sand that might be removed by the drawing of the model, then set the upper part or ring of the model firmly to its place and pour in the metal and allow it to cool. Then remove the ring, take out the model, attend to any defects that may arise from pouring the metal, set the cast on a bench, model upward, pin a slip of thick brown paper around the cast, pour the lead inside of the paper—the paper is strong enough to hold the lead without the support of sand—remove the paper and the cast and die are complete.

By this method the difficult casts can be made with the use of about a handful of sand, and with much less labor than by the ordinary method.—*Canada Journal of Dental Science.*

